# Has the “M” word been framed? Marijuana, cannabis, and public opinion

**DOI:** 10.1371/journal.pone.0224289

**Published:** 2019-10-31

**Authors:** Robert A. Mikos, Cindy D. Kam

**Affiliations:** 1 Law School, Vanderbilt University, Nashville, TN, United States of America; 2 Department of Political Science, Vanderbilt University, Nashville, TN, United States of America; University of the West of Scotland, UNITED KINGDOM

## Abstract

Over the past two decades, a growing cadre of US states has legalized the drug commonly known as “marijuana.” But even as more states legalize the drug, proponents of reform have begun to shun the term “marijuana” in favor of the term “cannabis.” Arguing that the “M” word has been tainted and may thus dampen public support for legalization, policy advocates have championed “cannabis” as an alternative and more neutral name for the drug. Importantly, however, no one has tested whether calling the drug “cannabis” as opposed to “marijuana” actually has any effect on public opinion. Using an original survey experiment, we examine whether framing the drug as “marijuana” as opposed to “cannabis” shapes public attitudes across a range of related topics: support for legalization of the drug, moral acceptance of its use, tolerance of activities involving the drug, perceptions of the drug’s harms, and stereotypes of its users. Throughout each of our tests, *we find no evidence* to suggest that the public distinguishes between the terms “marijuana” and “cannabis.” We conclude with implications of our findings for debates over marijuana/cannabis policy and for framing in policy discourse more generally.

## Introduction

Over the past two decades, a growing cadre of US states has legalized the drug Americans commonly refer to as “marijuana.” Although every state once prohibited the substance outright, more than 30 states now allow its use for medical purposes, and at least ten of those states allow use by adults for other purposes as well [1]. But even as more states legalize the drug, proponents of reform have begun to shun the term “marijuana.” As one lobbyist recently insisted, “We don’t use the ‘M’ word. It’s cannabis, cannabis, cannabis” [2], see also [3]. Indeed, the term “cannabis” is fast displacing “marijuana” in this policy space. For example, it appears in the names of recently formed interest groups such as the National *Cannabis* Industry Association, state-licensed businesses such as Green Man *Cannabis*, and trade publications like the *Cannabis* Business Executive.

Although “marijuana” and “cannabis” both refer to the same drug, reformers fear that the word “marijuana” has been indelibly marred by longstanding prohibitions that targeted the drug by that name. Until very recently, almost every state and the federal government referred to the drug as “marijuana” (or “marihuana”) rather than “cannabis” in their official legal codes [1]. As Gettman (2015) argues, “marijuana” was the term favored by prohibition supporters “as they sought to demonize its use and criminalize its consumers” [4]. Indeed, some claim that lawmakers originally adopted the foreign-sounding word “marijuana” “precisely because they wanted to underscore that it was a Latino, particularly Mexican ‘vice’” and thereby boost support among xenophobes for laws prohibiting the drug [5], [6], [7], [8], [9].

To its champions, the term “cannabis” carries no such baggage. “Cannabis” comes from the Latin word “cannabis” and the earlier Greek word “κάνναβις” [10] and is the scientific name given to the plant *Cannabis Sativa* and its variants from which the drug is produced. The origins of the word “marijuana” are murkier, but it likely derived from a Mexican-Spanish slang word or name [11], [12]. Thus, advocates hope that changing the name can boost public support for their reform proposals and for the people who consume and supply the drug [13], [14], [15], [16]. After all, if “marijuana” conjures up negative associations, then shedding that term for one that is ostensibly more neutral (like “cannabis”) should defuse opposition to their cause. The following call-to-arms from a prominent “cannabis” (née “marijuana”) distributor exemplifies the point:

… By changing the words we use to describe cannabis and herbal medicine, we can help our fellow citizens understand the truth about it, and see through the decades of propaganda.

That understanding will convert cannabis opponents into supporters, and bring closer the day when all our prisoners go free, and nobody else is ever again arrested for using or possessing ‘marijuana’. [17]

**Fig 1:**
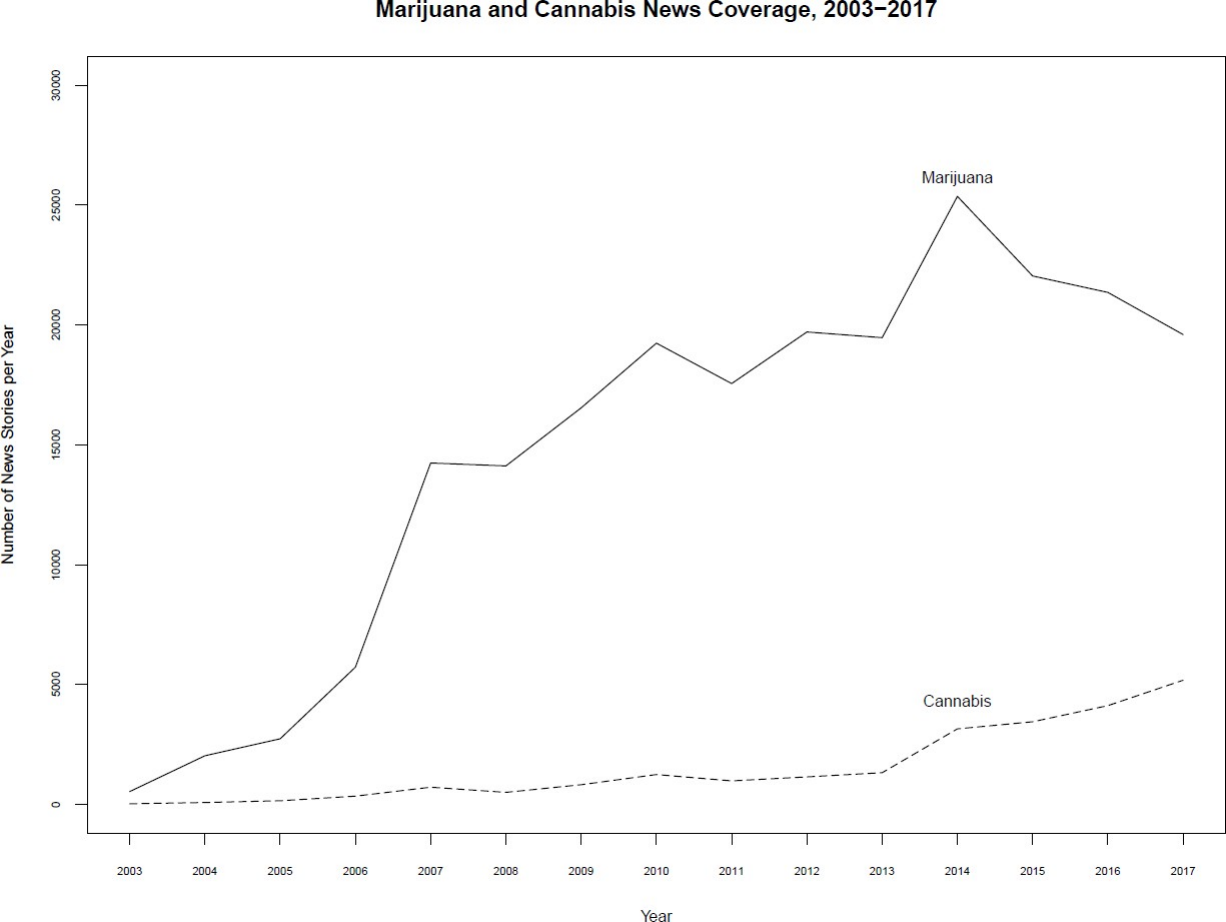
Mentions of “Marijuana” and “Cannabis” in US newspapers, 2003–2017. *Source*: Nexis-Uni searches of published stories in newspapers in the United States, Jan. 2003-Dec. 2017.

**Fig 2:**
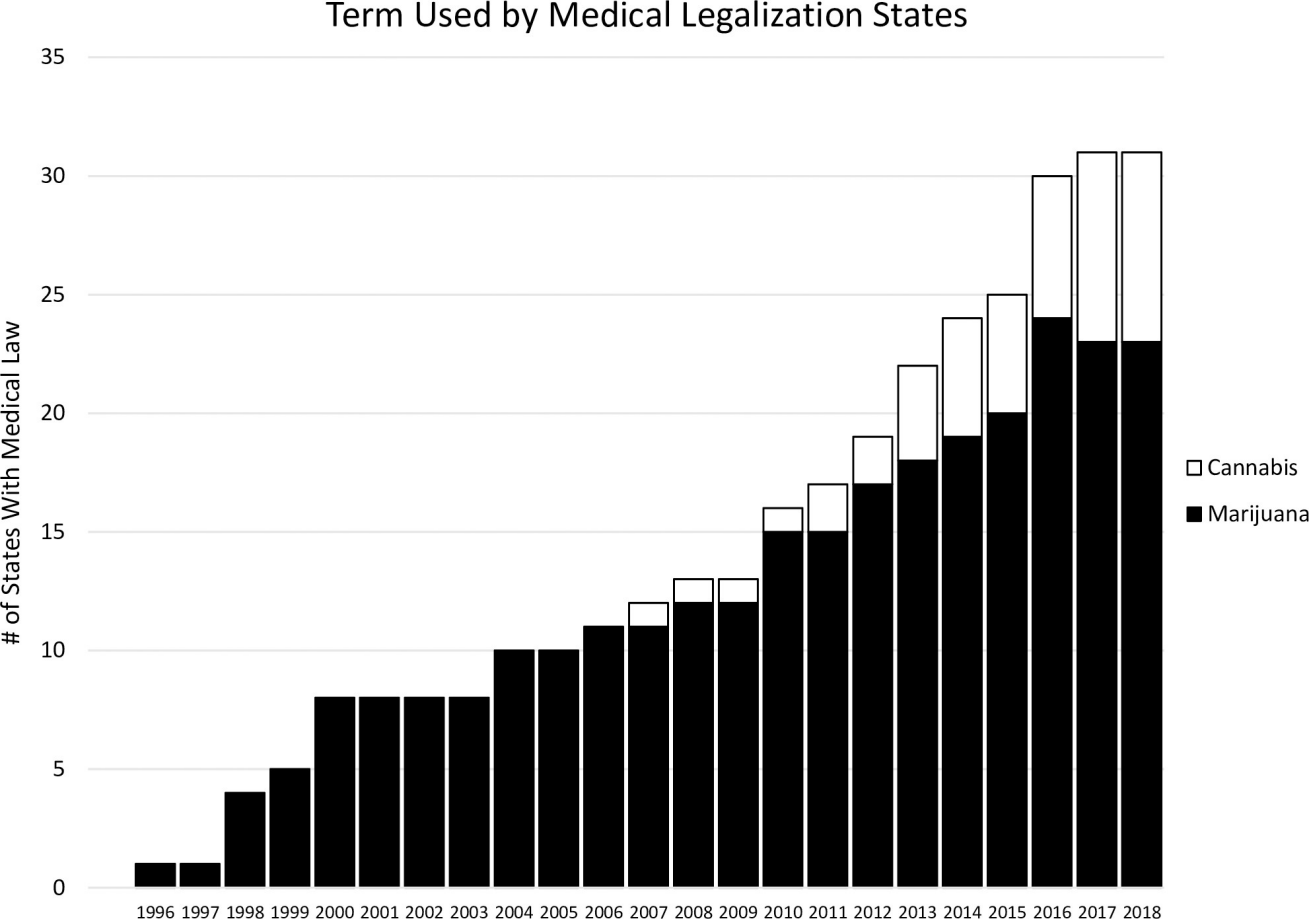
Terminology used in medical legalization states, 1996–2018. *Source*: Westlaw Edge searches of state legislation, 1996–2018.

**Fig 3:**
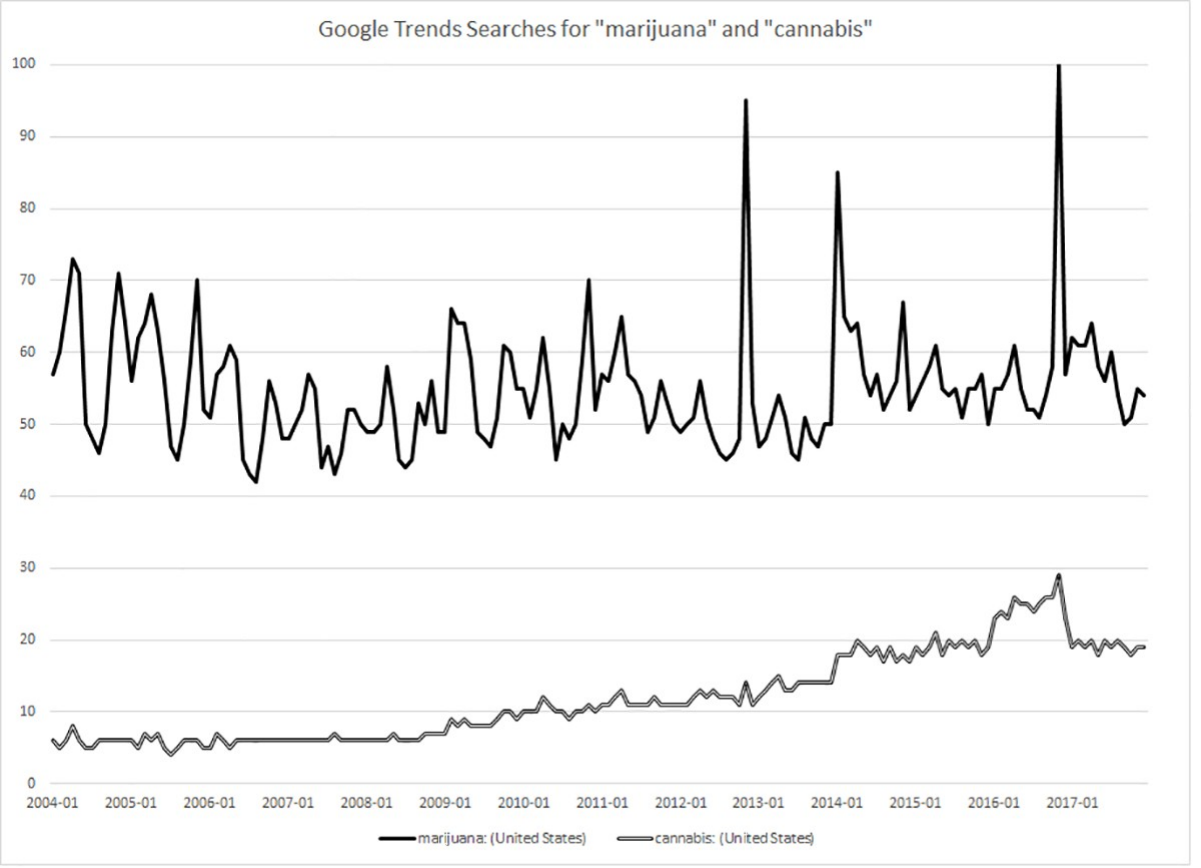
Relative popularity of “Cannabis” and “Marijuana” in Google searches in the United States, 2004–2017. *Source*: Google Trends query, June 22, 2018.

Advocates are clearly making headway in changing the terms used in public discourse. Trends in the media, state legislation, and internet searches all serve to demonstrate the shift. In the national media, where coverage of the drug has skyrocketed in the wake of recent reforms, “marijuana” is still the more commonly used of the two terms. But since 2014, “cannabis” has been swiftly gaining ground, as shown in Fig 1. Furthermore, while nearly every state once employed the label “marijuana” (or “marihuana”) in its legal code (as noted above), several states that have recently legalized the drug have also redubbed it “cannabis,” as shown in Fig 2. In similar fashion, “cannabis” has also been catching up to “marijuana” as a search term in Google, as shown in Fig 3.

All of this suggests that framing—and particularly, the choice between the terms “marijuana” and “cannabis”—has become part of the political debate surrounding this drug. The framing literature suggests that the words advocates use to discuss public policies can influence how the public thinks about those policies, including the factors individuals will consider when formulating their opinions [18]. In this vein, prior work has demonstrated that even slight changes in how policies are framed or named can affect public support for those policies. For example, Smith documents vast differences in public support for “assistance to the poor” as opposed to “welfare,” finding the latter is more likely to conjure up negative associations with “waste and bureaucracy” (p. 75 in [19]). Similarly, Schuldt et al. find not only that partisan elites selectively employ the terminology of “global warming” versus “climate change,” but also that these frames exacerbate the partisan divide in the general public’s beliefs about whether the phenomenon is occurring [20]. But it is just as important to recognize that alterations in framing and naming do not *always* make a difference. For example, Rudolph finds no difference in the level of support for cuts to the inheritance tax, whether it is framed as an “estate tax” or a “death tax” [21]. Furthermore, even small changes in terminology (such as “climate change” versus “global warming”) can affect different groups in ways that balance out in the aggregate [22]. Our work adds to this literature by examining how changes in terminology that have been championed by policy advocates may affect public opinion on another very salient political issue.

Frames can also conjure different connections between policies and social groups. Social groups play a critical role in shaping public opinion [23], [24], [25]. In this policy domain, no less than others, advocates have sought to associate their own causes with particular social groups in ways that they believe will bolster their prospects with lawmakers and the public. As discussed earlier, many have accused prohibitionists of exploiting xenophobia by calling the drug by a foreign-sounding name (“marijuana” or “marihuana”). Some have even accused modern-day reformers of co-opting this strategy. While not focusing on any of the frames discussed here, Schlussel argues that reformers have intentionally framed campaigns around themes of white individualism to garner majority support for marijuana legalization [26].

Importantly, however, no one has yet demonstrated that changing the terms of discourse—i.e., calling the drug “cannabis” as opposed to “marijuana”—actually has any effect on public opinion. Broadly available survey data from prominent survey houses provide no way of systematically testing the effects of the different terms. A search of the Roper Center’s iPoll database (the most comprehensive database of US public opinion polling) returned 452 survey questions between 2003–2017 including “marijuana,” but not a single one including “cannabis”. At most, the existing data demonstrate some related framing effects when the drug is labeled as “marijuana”: support for legalization of “*medical* marijuana” outstrips support for legalization of “*recreational* marijuana” by a wide margin [27] and legalization of marijuana for “medicinal treatment” outstrips support for legalization for “recreational use” [28].

In this article, we employ a novel experimental survey design to test whether the choice of the term “marijuana” versus the term “cannabis” affects public opinion toward the drug and the policies governing it, both for medical use and for use more generally. While other slang terms for the drug exist, we choose to focus on “cannabis” and “marijuana” because none of these other terms has been similarly championed or vilified by policy advocates. Based on evolving public discourse, as well as the arguments advanced by advocates, we hypothesize that public attitudes toward the drug and liberalization of the policies governing it should be more negative when the drug is referred to as “marijuana” rather than “cannabis.” We call this the *name frame hypothesis*. These changes may be attributable to the different considerations that people bring to mind when prompted by the different names for the drug. For one thing, the terms “marijuana” and “cannabis” may elicit different stereotypes of the people who use the drug. It is also possible that “cannabis” is a less familiar term for the drug. Calling it “cannabis” may thus induce some confusion among respondents, in which case we would expect to see greater incidence of “Don’t know” responses to survey questions. We call this the *unfamiliarity hypothesis*. We apply these hypotheses to a broad set of dependent variables in this policy domain, including public opinion on legalization, evaluations of morality, tolerance of different activities involving the drug, perceptions of harm, and stereotypes of users.

After a discussion of Methods and Results, we close with a discussion of the ramifications of our findings for debates over policies toward this drug and for framing in policy discourse more generally. We also consider some possible alternative justifications for changing the name apart from marshalling public support for reforms. For example, one justification is that the term “marijuana” is offensive to some because it is associated with racist campaigns that pushed for prohibition [29].

## Methods

In August 2017, we contracted with YouGov to survey 1,600 adults who are part of their standing panel of online survey respondents. The data, when weighted, are nationally representative of the English-speaking adult general population in the United States. Table 1 provides descriptive statistics for the sample. Protocols were approved by the Vanderbilt University Human Research Protections Program: IRB#171304, and the data were anonymized by YouGov before delivery to the researchers.

**Table 1:** Sample demographics, weighted analysis.

Characteristic	Sample
Female	51.8%
Mean Age (s.e.)	40 (0.01)
Nonwhite	27.2%
Education:	
No High School	12.3%
High School Degree	30.2%
Some College	19.1%
Associate Degree	12.5%
College Degree	16.7%
Post-Graduate Degree	9.3%
Region:	
Northeast	19.6%
South	34.8%
Midwest	21.9%
West	23.7%
Partisanship	
Democrat	32.5%
Independent	38.8%
Republican	23.9%
Not sure	4.8%

Each respondent was randomly assigned to one of four conditions in which we experimentally varied the term used in each of our questions to be “marijuana,” “cannabis,” “medical marijuana,” or “medical cannabis.” Each of our questions embedded that randomly assigned drug term consistently throughout the survey. After consenting to participate in the survey, respondents were asked background questions and then a series of questions to gauge their opinions on a broad range of topics related to the drug. Table 2 provides the question text for each of the questions.

**Table 2:** Question text and response options.

Topic	Question Text	Response Options
Legalization	Do you think the use of [X] should be legal or not?	Strongly support legalization Somewhat support legalization Don’t know/not sure Somewhat oppose legalization Strongly oppose legalization.
Moral acceptability	Regardless of whether you think [X] should be legal or not, do you believe that, in general, using [X] is morally acceptable [wrong] or morally wrong [acceptable]?	[response options rotated to match question text] Morally acceptable Don’t know Morally wrong
Tolerance of drug activities	If [X] were legal, how would you feel if: …a store or business selling [X] opened up in your neighborhood? …people used [X] in public places like parks and restaurants? …a public school teacher used [X] during non-school hours?	Would bother me a lot Would bother me somewhat Would bother me a little Would not bother me at all
Perceptions of harm	Do you agree or disagree with the following statements: The use of [X] is addictive. The use of [X] leads to the use of other drugs. The use of [X] harms the user’s health. The use of [X] significantly impairs driving.	Strongly agree Somewhat agree Not sure/Don’t Know Somewhat disagree Strongly disagree
Stereotypes of users	How well does each of these words describe people who use [X]? [randomize order]: Lazy, Hard-working, Healthy, Sick, Honest, Opportunistic, Teenagers, Elderly, Blacks, Latinos, Whites, Irresponsible, Dependable, Rich, Poor	Extremely well Very well Moderately well Slightly well Not at all

[X]: Respondents are randomly assigned to questions containing “marijuana”, “cannabis”, “medical marijuana”, or “medical cannabis” for the duration of the survey.

Balance tests for the “marijuana” vs. “cannabis” comparisons (overall and by medical vs. unspecified use) showed no significant relationships between treatment assignment and demographic covariates (sex, age, education, race, region, or partisanship). Analyses are conducted with ordinary least squares (OLS) regression with survey weights applied to estimate Average Treatment Effects (ATEs) without covariate adjustment.

## Results and discussion

### Legalization

**Fig 4:**
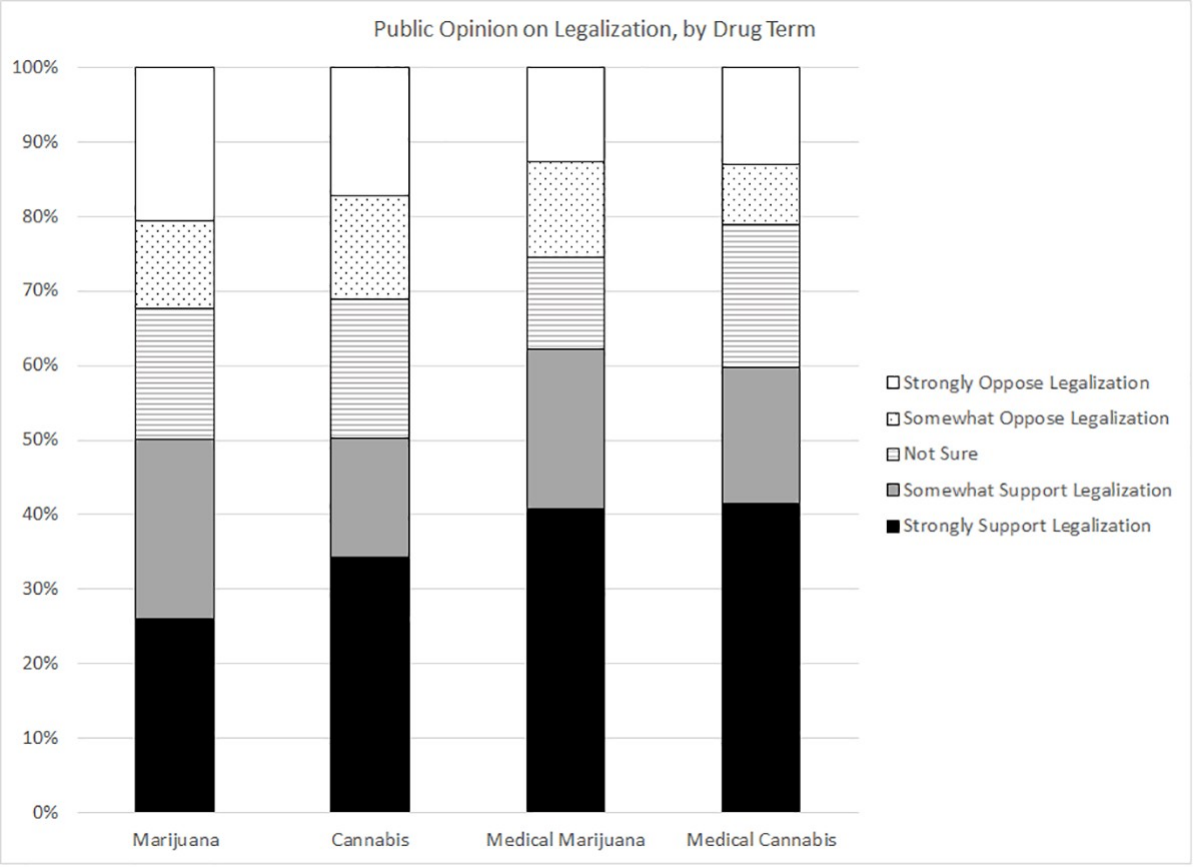
Public opinion on legalization by drug term.

We begin by examining the distribution of opinion on legalization (shown in Fig 4), focusing initially on what we consider the baseline condition, “marijuana.” A bare majority (50.1%) of respondents favor “marijuana” legalization (with 26.0% strongly favoring and 24.1% somewhat favoring). About a third (32.4%) of respondents oppose legalization (20.6% strongly opposing and 11.8% somewhat opposing). A sizable proportion (17.5%) select the “Don’t know/not sure” option. Setting aside those who select the “Don’t know/not sure” option, 61% favor legalization and 39% oppose it; these percentages compare very favorably to existing survey benchmarks [30], [31].

Fig 4 depicts descriptive differences across the conditions. The distribution of opinion towards “marijuana” and “cannabis” is quite similar—overall 50.1% and 50.3%, respectively, support legalization of the drug, although there is a slight uptick in intensity of support for legalization of “cannabis”—34.3% strongly support legalization of the drug by that name, whereas only 26.0% register such enthusiasm for the legalization of “marijuana.” Fig 4 also shows that support for legalization is higher when the “medical” label is attached to the drug, whether it is called “medical *marijuana*” or “medical *cannabis*” (62.2% and 59.7%, respectively).

To subject these comparisons to statistical tests, we model public opinion on legalization (coded from 0 to 1 with higher values indicating stronger support) as a function of: a dummy variable for *cannabis* (coded 1 if the drug is referred to as “cannabis” or 0 if “marijuana”), a dummy variable for *medical* (coded 1 if the usage is specified as “medical” or 0 if not specified), and their interaction. In our main analyses, we set “Don’t know/not sure” responses to the midpoint. Omitting these responses produces statistically and substantively similar results. Due to its more straightforward interpretation, we use OLS regression (results with ordered probit are substantively and statistically similar). The OLS results appear in Table 3.

**Table 3:** Regression analysis of policy opinions and moral /tolerance/ safety evaluations.

	**Legalization**	**Moral** **Acceptability**	**Tolerance:** **Store**	**Tolerance:** **Public Places**	**Tolerance:** **Off-Duty Teacher Use**	**Not** **Addictive**	**Not a Gateway**	**Not Harmful to Health**	**Does Not Impair Driving**
**Cannabis**	0.03	0.03	-0.02	-0.02	-0.01	0.02	0.01	0.03	0.01
	0.04	0.04	0.04	0.04	0.04	0.04	0.04	0.03	0.03
**Cannabis**	-0.03	-0.01	0.05	0.05	0.00	-0.07	-0.05	-0.06	-0.01
**X Medical**	0.05	0.05	0.05	0.06	0.06	0.05	0.05	0.04	0.05
**Medical**	0.10***	0.13***	0.04	0.08*	0.11***	0.07**	0.09***	0.15***	0.08***
	0.03	0.04	0.04	0.04	0.04	0.04	0.04	0.03	0.03
**Intercept**	0.56	0.58	0.62	0.43	0.57	0.46	0.50	0.40	0.28
	0.02	0.03	0.03	0.03	0.03	0.02	0.03	0.02	0.02
**N**	1591	1597	1592	1548	1587	1544	1598	1591	1598

Baseline reference group is “Marijuana” condition.

Dependent variables coded with higher values indicating more support for legalization, higher levels of acceptability and tolerance, and lower levels of belief in harms.

Table entry is OLS regression coefficient with standard error below. Survey weights applied.

*p<0.10

**p<0.05

***p<0.01

two-tailed.

Contrary to expectations, we find no support for the name frame hypothesis: average opinion on “marijuana” legalization is *not* statistically distinguishable from average opinion on “cannabis” legalization. In other words, calling the drug “cannabis” does not boost public support for legalization of the drug. Not only is the effect statistically insignificant, but it is substantively tiny (b = .03, *p*~0.37). Likewise, the name of the drug does not matter when we specify that is only for “medical” use. To be sure, we find that the public does distinguish between medical use of the drug and use more generally, as shown by the significant coefficient (b = 0.10, *p*<0.01) on *medical*. But the name of the drug itself does not contribute to these differences (as shown by the insignificant and tiny coefficient on the interaction term).

Separate from affecting the direction of support for legalization, we also hypothesized that calling the drug “cannabis” might sow uncertainty among respondents who are unfamiliar with the term, thereby generating a relatively high rate of “Don’t know/not sure” responses in the two “cannabis” conditions. To test this unfamiliarity hypothesis, we examine the proportion of respondents who elect the “Don’t know/not sure” option across conditions. Using the same model above, we find no statistically significant differences in familiarity attributable to the name frame (or to specified medical usage): *cannabis* carries a substantively tiny (b = 0.01) and statistically insignificant (*p*~0.82) coefficient, *medical* depresses rates of “Don’t Know” by about five percentage points (but this difference is not statistically distinguishable from zero, *p*~0.19), and the interaction between *cannabis* and *medical* is not significant (b = 0.06, *p*~0.29).

In short, we find no support for the hypothesis that calling the drug “cannabis” as opposed to “marijuana” will boost public support for its legalization, whether for medical use or use more generally. The rates of “Don’t know/not sure” do not shift significantly with terminology either, providing little evidence supporting the unfamiliarity hypothesis.

### Opinion on other aspects of the drug

To provide additional tests of our hypotheses, we asked respondents questions about several topics besides legalization, including moral acceptability of the drug, tolerance of drug-related activities, perceptions of the drug’s harms, and stereotypes of its users.

### Moral acceptance

We asked respondents about their moral acceptance of using the substance (however named), setting aside the question of the drug’s legality. Histograms by experimental treatment appear in S1 Fig.

In our initial descriptive review of the distributions, the percentage of respondents who find use of “marijuana” and “cannabis” morally *acceptable* is quite similar (43.8% versus 44.3%). There is a slight difference in the percentage of respondents who find “marijuana” use morally *wrong* relative to “cannabis” use (27.2% versus 20.8%). Moral acceptance of use of the drug appears to be higher when the label “medical” is attached to it–whether it is attached to “marijuana” (58.6%) or “cannabis” (61.7%). Likewise, moral disapproval appears to be lower when use is for medical purposes: only 15.3% and 12.9% of respondents believe it is morally wrong to use “medical marijuana” or “medical cannabis,” respectively.

The regression analyses in Table 3 subject these comparisons to statistical tests. We again find little evidence to support the name frame hypothesis. The coefficient on *cannabis* is small and statistically indistinguishable from zero, as is the interaction term between *cannabis* and *medical*. As with legalization, the public does distinguish between medical and unspecified use of the drug. But again, we find that these differences hold regardless of the name (marijuana or cannabis) given the drug. In tests of the unfamiliarity hypothesis, we again uncover no statistically distinguishable effects attributable to the experimental conditions, thus providing little evidence to support the unfamiliarity hypothesis.

### Tolerance of drug activities

We next asked respondents about their tolerance toward three activities that could follow legalization of use: opening of a store in the respondent’s neighborhood, public use of the drug, and off-duty use by a public school teacher. Histograms by experimental treatment appear in the S2 Fig.

Comparing the statistics descriptively, respondents are most bothered by use in a public place (aggregating across terms, 72.9% said they would be bothered a lot, somewhat, or a little by it) and less bothered by the prospect of a store opening in their neighborhood (52.0%) or a public school teacher using it off-duty (52.7%). For each scenario, however, no appreciable difference in tolerance emerges between the two names given the drug: roughly the same percentage of respondents are bothered (to at least some degree) by the opening of a nearby store, public use, and off-duty use by a teacher, when we call the drug “marijuana” as when we call the drug “cannabis.” As with our other dependent variables, responses appear to be more positive when medical use is specified. Stated differently, the public is less bothered by each of these scenarios when “medical marijuana” or “medical cannabis” is involved than when “marijuana” or “cannabis” is involved.

In OLS regression analyses (Table 3), we find no statistically distinguishable effects on any of the questions when comparing “marijuana” with “cannabis”—i.e., there is no support for the name frame hypothesis, although in two of the questions, we do see enhanced tolerance for medical use compared to when the purpose of usage is left unspecified.

### Potential harms

One contested part of the debate over (medical) marijuana/cannabis legalization concerns the potential harms of the drug. We asked respondents to register their level of agreement or disagreement with attributing four potential harms to the drug: *that it is addictive*, *leads to the use of other drugs*, *harms the user’s health*, and *significantly impairs driving*. Histograms by treatment condition appear in S3 Fig.

A descriptive review of the data indicates three noticeable patterns. First, the public appears more convinced that the drug impairs driving than that it causes the other three harms (addiction, use of other drugs, and damage to user health). Second, for all four of the harms, there is no noticeable difference when we compare responses in the “marijuana” and “cannabis” conditions. About the same percentage of respondents think the drug is addictive, leads to the use of harder drugs, harms user health, and impairs driving when we call it “cannabis” as when we call it “marijuana.” Third, views of the drugs’ harms do appear to differ when respondents are told it is to be used only for medical purposes. In other words, the public views the drug (whether we name it “marijuana” or “cannabis”) to be less harmful—less addictive, less likely to lead to use of other drugs, less damaging to user health, and less likely to impair driving—when it is designated as “medical.”

In regression analyses (Table 3), we find no statistically significant differences between the public’s views of the harms associated with “marijuana” and its views of the harms associated with “cannabis.” We again find that focusing only on medical use does have a demonstrable impact on opinion. In particular, attaching the medical label to the drug’s name appears to allay concerns about each of the drug’s purported harms. In the conclusion, we discuss possible explanations for why the public may deem the drug less harmful when told that its use is limited to medical purposes.

We uncover little evidence supporting the unfamiliarity hypothesis in assessments of the drug’s harms. There are no statistically significant differences in rates of “Don’t know/not sure” between “marijuana” and “cannabis”, and there are no statistically significant differences between “medical marijuana” and “medical cannabis.”

### Stereotypes of users

To investigate whether the linguistic frames used in debates over the drug evoke different stereotypes of users, we developed a novel instrument to gauge the degree to which individuals attribute given characteristics relating to membership in demographic groups, vice, virtue, and health status to people who use the drug. Histograms by treatment condition appear in S4 Fig.

While there is some variability in the traits associated with these terms, responses to the two unmodified terms (“marijuana” and “cannabis”) seem to cluster together and responses to the two medically focused terms (“medical marijuana” and “medical cannabis”) cluster together. Digging into the specifics, four terms are among the five characteristics most highly associated with *both* “marijuana” and “cannabis” users: “Teenaged,” “White,” “Irresponsible,” and “Lazy.” “Sick” rounded out the top five in the “marijuana” condition (fifth), as did “Black” in the “cannabis” condition (third). The terms that are *least* associated with these users are “Elderly,” “Opportunistic,” “Latino,” and “Healthy” for “marijuana,” and “Elderly,” “Opportunistic,” “Healthy,” and “Rich” for “cannabis.” For “medical marijuana” and “medical cannabis” users alike, the five most highly associated traits are the same (albeit in slightly different rank ordering): “Sick,” “Honest,” “White,” “Hardworking,” and “Dependable.” Similarly, the terms that are least associated with both “medical marijuana” and “medical cannabis” users are the same (again, in somewhat different rank order): “Irresponsible,” “Lazy,” “Poor,” and “Healthy.”

**Fig 5:**
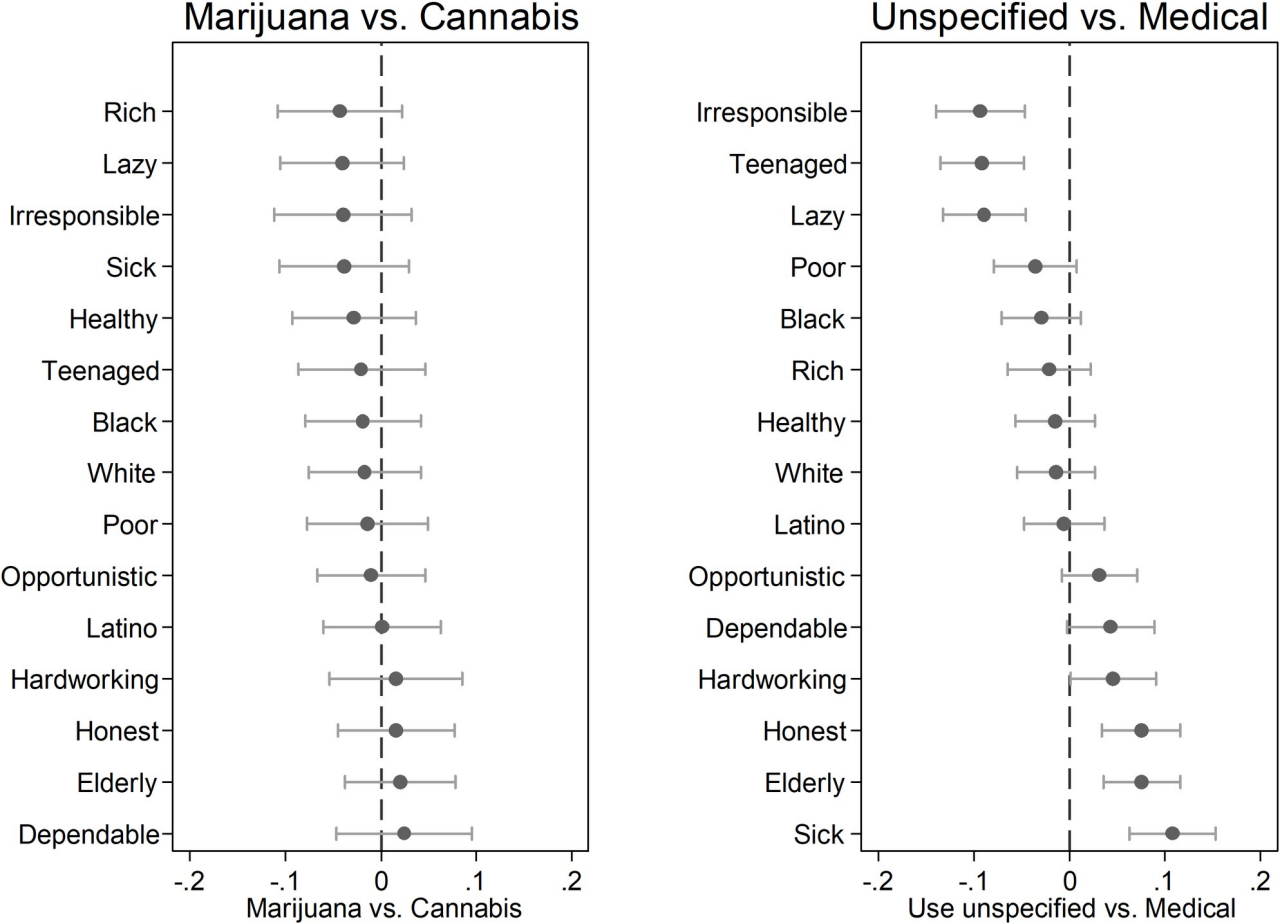
Traits of users, regression coefficients and confidence intervals. OLS regression coefficients with 95% confidence intervals. Survey weights applied. Marijuana vs. Cannabis comparison reflects unspecified use. Unspecified vs. Medical comparison aggregates marijuana with cannabis in each use frame.

We used regression analysis to estimate statistical differences in how closely respondents associate users with each characteristic, by drug term. For visual compactness, we display the coefficients with their 95% confidence intervals in Fig 5. Importantly, we find *no* statistically significant difference for *any* of the fifteen characteristics when we compare responses in the “marijuana” and “cannabis” conditions. We also find no statistically significant differences when we compare responses to these two terms in the medical conditions (i.e., “medical marijuana” and “medical cannabis”). We do, however, turn up several statistically significant differences when we compare the terms from the medical use conditions with the terms in the unspecified use conditions: the traits “Irresponsible”, “Teenaged”, and “Lazy” are more associated with users of marijuana/cannabis than with users of medical marijuana/medical cannabis (*p* < 0.01 in each model). The traits “Hardworking,” “Honest,” “Elderly,” and “Sick” are more associated with medical marijuana/medical cannabis than marijuana/cannabis (*p*<0.05 for the first and *p*<0.01 for the last three models).

### Summary of results

**Fig 6:**
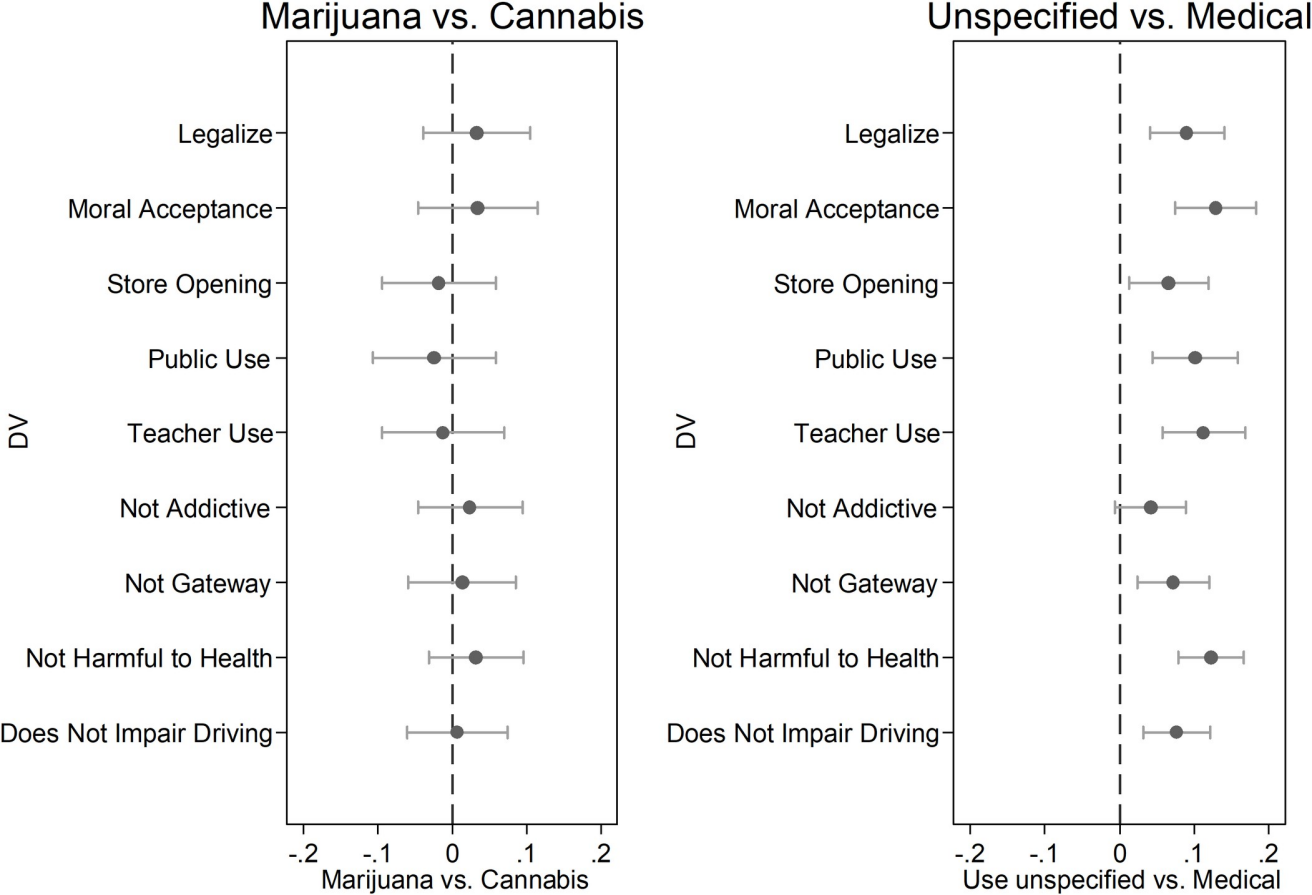
Summary of findings on policy and evaluations. OLS regression coefficients with 95% confidence intervals. Survey weights applied. Marijuana vs. Cannabis comparison reflects unspecified use. Unspecified vs. Medical comparison aggregates marijuana with cannabis in each use frame.

Fig 6 collects and summarizes all of our comparisons and makes clear that in each and every test, the name frame (“marijuana” versus “cannabis”) has no impact on opinion toward the drug. Our results thus undermine the notion—widely espoused by policy advocates—that abandoning the word “marijuana” for “cannabis” by itself will boost the prospects for reform or soften public attitudes toward the drug.

## Conclusions

Our novel survey experiment provides systematic evidence on the extent to which framing in political discourse affects public opinion toward the drug commonly known by Americans as “marijuana.” We find no support for the notion that changing the name of the drug from “marijuana” to “cannabis” affects public opinion on the drug or the policies governing it. Whether asked about legalization of the drug, the moral acceptability of its use, tolerance for activities involving the drug, the harmfulness of its use, or the traits of its users—and whether they are prompted to think about medical use or use more generally—respondents offered similar opinions whether we called the drug “marijuana” or “cannabis.” Given our sample size of 400 respondents per condition, the survey is sufficiently powered to detect even a small effect size (Cohen’s *d* = 0.2), holding α = .05 and power = 0.8. Thus, the lack of any meaningful difference in responses to the two names is not simply a feature of low statistical power, as the point estimates we report are not simply statistically insignificant: they are *substantively tiny*.

With these findings in mind, our study does have limitations. The results may be time-bound: as public discourse evolves and increasingly relies upon the use of “cannabis” vs. “marijuana,” the latter term could indeed become outdated and associated with the policies and attitudes of a prior era. Further, it is clear that our results are geographically-bound to the United States, given our respondents were from the United States and the usage of the term “marijuana” is peculiarly American. Finally, we note that this is a study of public opinion, not behavior: thus, we cannot know if framing would affect any individual’s decision to use, purchase, or otherwise engage in activities involving the drug.

More broadly, this article suggests that claims that framing will necessarily change public opinion toward the drug should be met with healthy skepticism. Advocates have pushed for abandoning the term “marijuana” in favor of “cannabis” at least in part to rally public support for reforms and for the people participating in them, without having firm evidence that the name change will actually produce the sought-after effect. Indeed, advocates have pushed for other changes in terminology surrounding this drug–for example, preferring the label “adult use” over “recreational use” (to describe non-medical laws), and preferring the label “consumer” to “user” (to describe those who put the drug in their bodies)—ostensibly based on a similar hope that the change in terminology could boost public support [32], [33], [34]. Investigation of these other frames might be warranted; our study was not designed to test these specific frames. Nonetheless, our results should give proponents some pause. Not every frame changes the picture.

Why have policy advocates misjudged public attitudes toward the “M” word? One possibility is that the term “marijuana” once actually did conjure up special negative associations, but that the word has since shed those associations. Although we cannot test this hypothesis with our data, historical polling shows that public attitudes toward “marijuana”—and, as noted in the Introduction, past surveys consistently used *that* term—have liberalized over time [35]. For example, the Gallup Organization has measured attitudes toward legalization of “marijuana” every year since 1969. In that first year, only 12% of respondents supported legalization of the drug, but by mid-2018, 66% of respondents supported legalization. The lesson may be that the associations people attach to particular words may change over time [36].

While advocates may be mistaken in thinking that a name change will redirect public opinion, there may be other reasons that justify the change in terminology. Here we consider two possibilities.

The first possibility is that the term “marijuana” is offensive to some, given the nefarious reasons some believe the drug was so labeled. In 2017, for example, when the Hawaii legislature passed a law that substituted “medical cannabis” for “medical marijuana” in all state statutes, it declared that whereas “‘Marijuana’ … carries prejudicial implications rooted in racial stereotypes, … the term cannabis carries no such negative connotations” [37]. This provides a moral justification for abandoning the term, regardless of whether it has any impact on public opinion—akin to the justification for the change of term for social groups such as African Americans (among others) in public discourse [38], [39], [40]. Put another way, while the public might not view the drug or the policies governing it any differently if it is called “cannabis” instead of “marijuana,” an intentional decision to use the name “cannabis” over “marijuana” could be justified on moral grounds.

The second possible rationale for the name change stems from globalization. The United States is exceptional in referring to the drug as “marijuana.” “Cannabis” is the more widely used term elsewhere in the world [41]. Google Trends data demonstrate this American exceptionalism. On August 22, 2019, we queried Google Trends and downloaded the comparison of “marijuana” with “cannabis” worldwide and by country for the ten-year time period between January 1, 2009 to December 31, 2018. In that ten-year period, “cannabis” had nearly overtaken “marijuana” in Google Trends worldwide. In the United States, searches reflect a 3:1 ratio: 77% are searches for “marijuana” and 23% for “cannabis.” In the United Kingdom, for example, the pattern is more than reversed: 84% of searches are for “cannabis” and 16% for “marijuana.” In Canada, the ten-year period reflects a fairly balanced ratio (56% of searches are for “marijuana” and 44% for “cannabis”), but when we restrict the time period to 2018 only, we find that “cannabis” has overtaken “marijuana” in search, with the former accounting for 63% of searches. The widespread use of the word “cannabis” outside the United States might exert some pressure—for business, cultural, scientific, or other reasons—for the United States to follow suit.

Even though the name attached to the drug appears to have no influence on public opinion, we find consistent support for the notion that the public views the drug more favorably when told it is for medical versus unspecified purposes. The public is much more supportive of legalization of medical use, more morally accepting of it, less bothered by activities involving it, less convinced that it is harmful, and more likely to attribute positive traits to its users when told that the drug is “medical.” While medical use appears to enjoy a halo effect—i.e., the public is generally more accepting of the drug when told it is for medical purposes and less concerned by its harms—that halo effect is the same regardless of whether we call the drug “medical marijuana” or “medical cannabis.”

Although our findings are consistent with the conventional wisdom regarding support for legalization for medical purposes [27], the finding that “medical marijuana”/“medical cannabis” is perceived as *less harmful* is somewhat puzzling. It is, after all, the same substance, whether it is used for medical or other purposes; it is just the purpose to which it is put that we vary in our question text.

Here, we suggest three possible explanations for this result. The first is that the public might think that people who use marijuana/cannabis for medical purposes generally do so more responsibly than do those who use the same drug for other purposes. Indeed, this explanation seems quite plausible given our finding that the public associates very different traits with medical than with non-medical users of the drug. They have sick, honest, and elderly people in mind when they think of users of medical marijuana and medical cannabis. They have teenaged, irresponsible, and lazy people in mind when they think of the users of marijuana and cannabis without the medical designation attached. The second possible explanation is that people believe (rightly or wrongly) that medical use, unlike, say, recreational use, is medically supervised in a way that might forestall some of the harms of unsupervised use. In states that have legalized medical marijuana/medical cannabis, for example, a physician must first recommend the drug to a patient before the patient may legally use the drug [1]. The third possibility is that people do not think the drug is the same when denoted for medical purposes. Some states do in fact limit how the drug may be consumed by medical patients, for example, banning edible or smokeable forms of the drug [1], and the public might view these limits as promoting safer consumption. Thus, our results may simply show that for the time being, the public prefers modest over bold reforms of the laws governing the drug, whether it is called “marijuana” or “cannabis.”

## Supporting information

S1 Fig Morality assessments by drug term. (TIF)

S2 Fig Tolerance of drug related activities by drug term. (TIF)

S3 Fig Beliefs about harms by drug term. (TIF)

S4 Fig Average traits of users, by drug term. (TIF)
