# Supplementary figures and images for: Has the “M” word been framed? Marijuana, cannabis, and public opinion
Source: PLoS One. 2019 Oct 31;14(10):e0224289. doi: 10.1371/journal.pone.0224289 (PMC6822944; doi:10.1371/journal.pone.0224289)

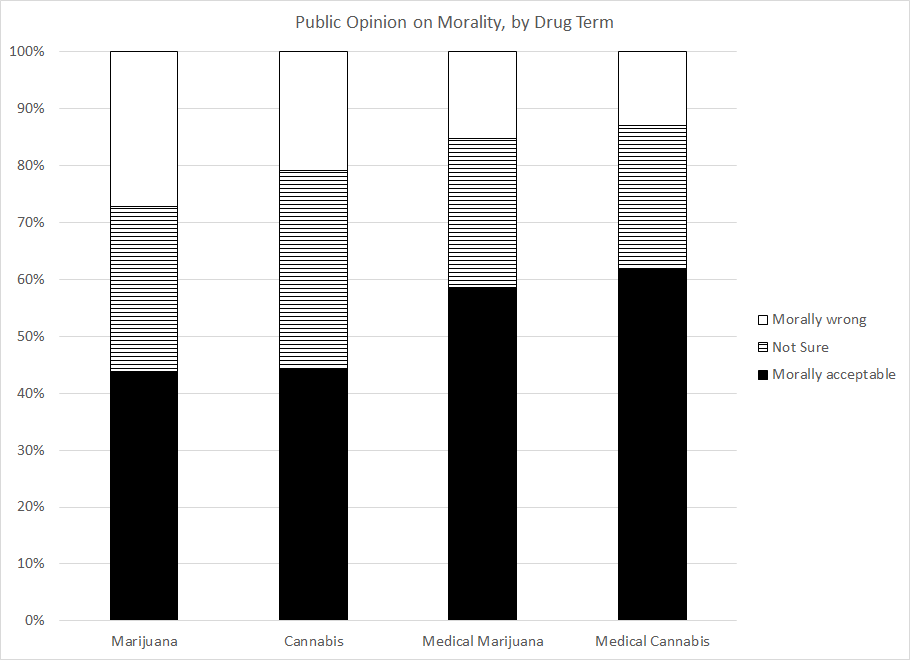

Supplement: S1 Fig — (TIF) [file pone.0224289.s001.tif]

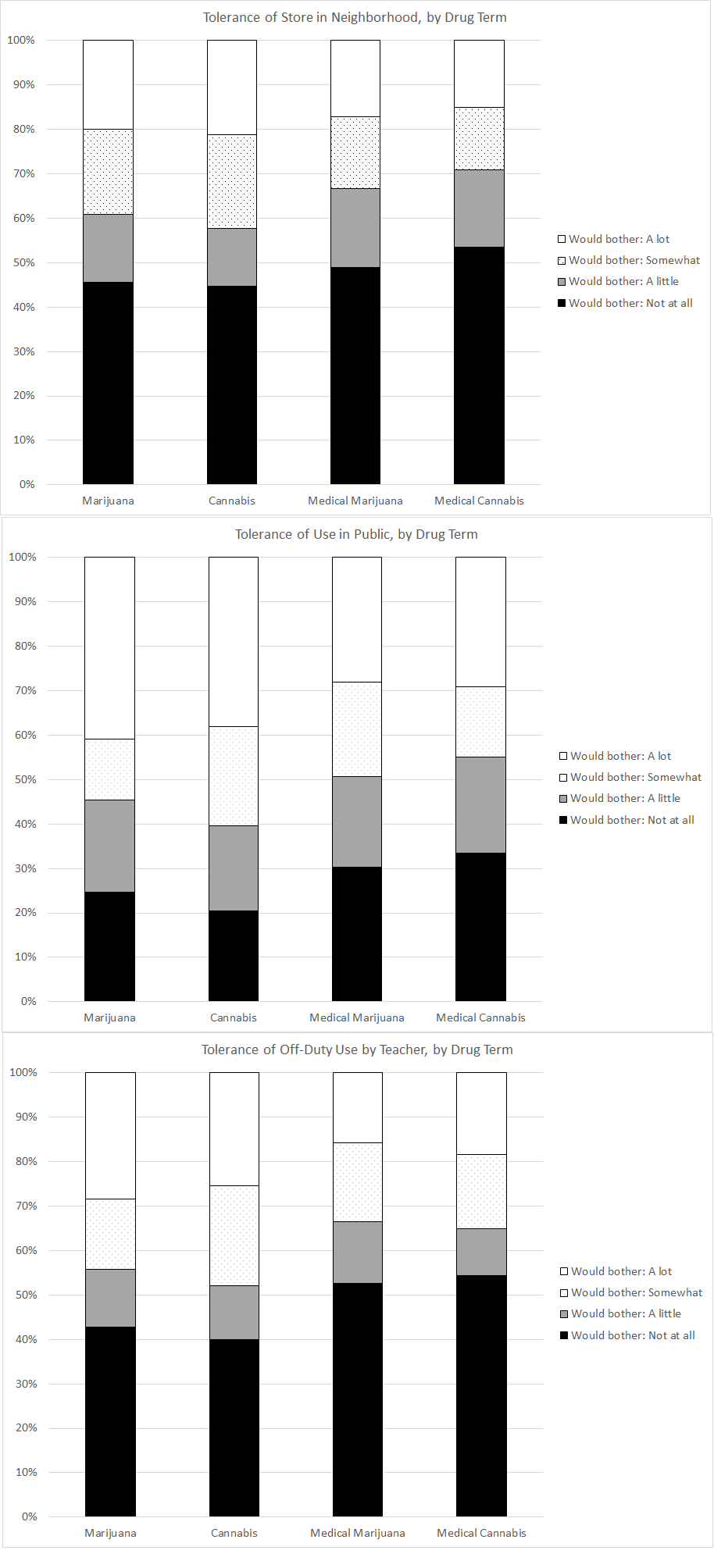

Supplement: S2 Fig — (TIF) [file pone.0224289.s002.tif]

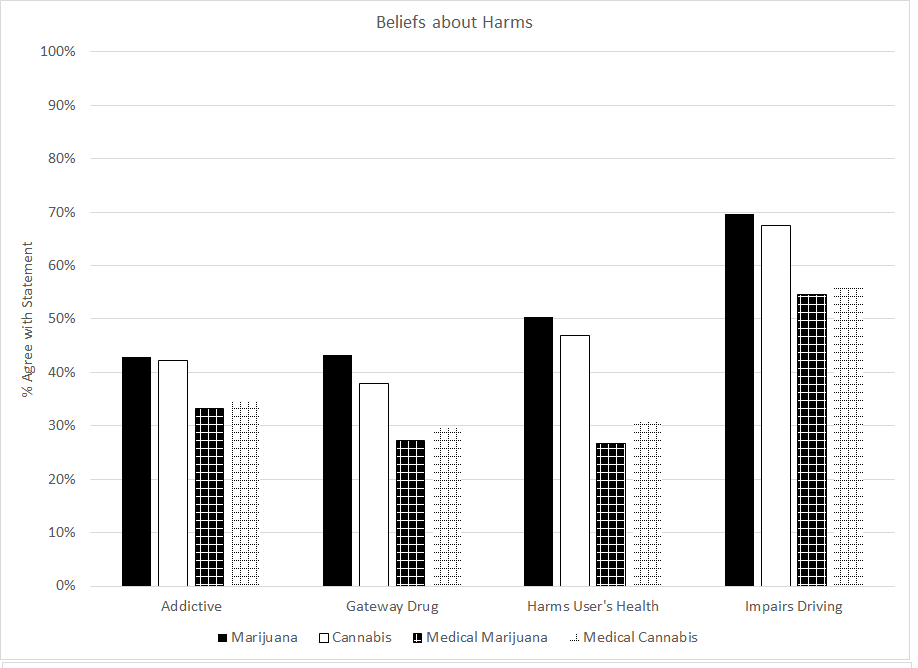

Supplement: S3 Fig — (TIF) [file pone.0224289.s003.tif]

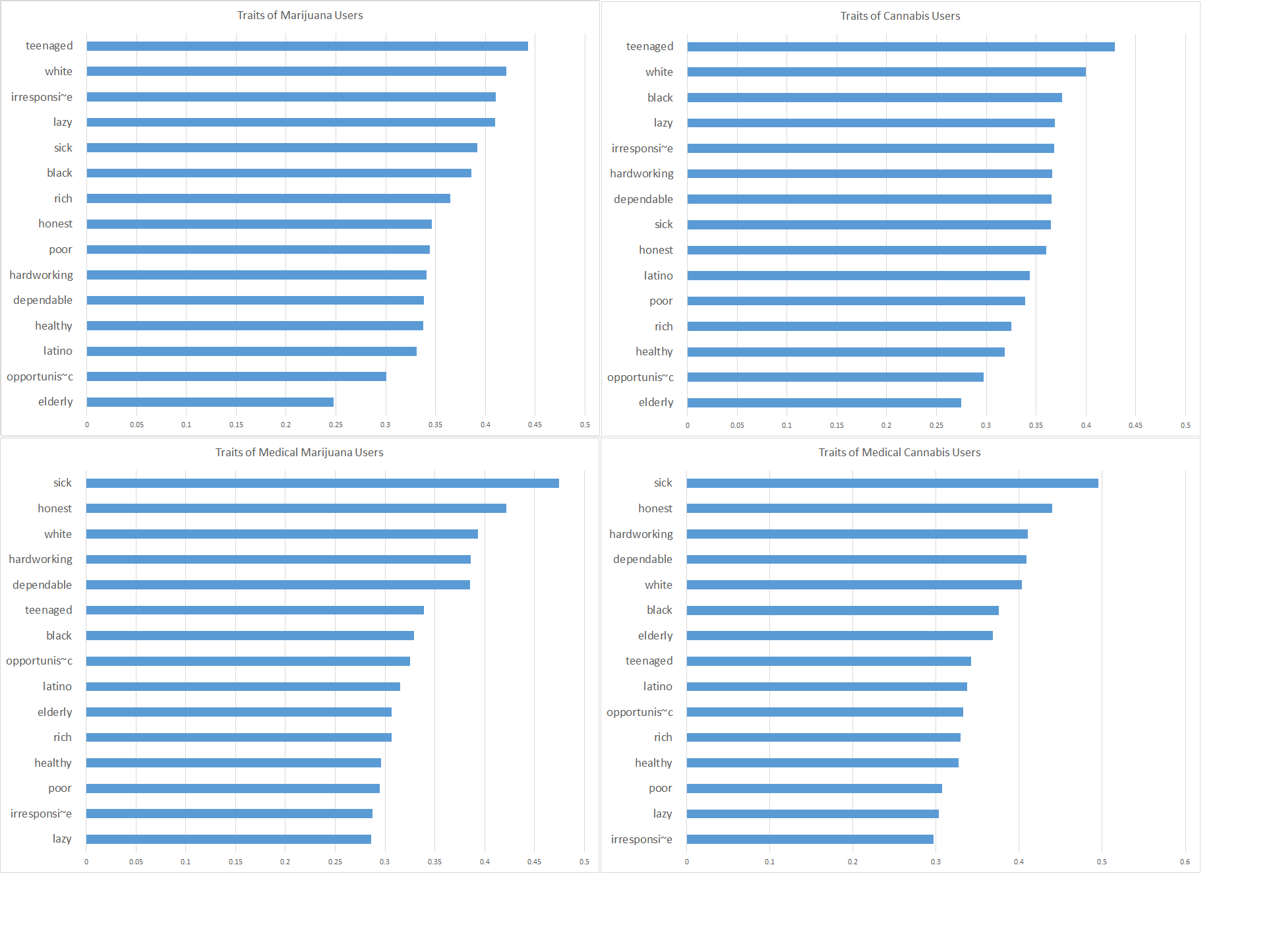

Supplement: S4 Fig — (TIF) [file pone.0224289.s004.tif]
